# Spatial network characteristics and influencing factors of residential land prices under the background of coordinated development: A case study of the Wuhan metropolitan area in China

**DOI:** 10.1371/journal.pone.0325946

**Published:** 2025-07-10

**Authors:** Wenqi Li, Fengjuan Wei

**Affiliations:** 1 School of Civil Engineering and Environment, Hubei University of Technology, Wuhan, China; 2 Innovation Demonstration Base of Ecological Environment Geotechnical and Ecological Restoration of Rivers and Lakes, Wuhan, China; Huazhong University of Science and Technology, CHINA

## Abstract

Exploring the spatial structure of residential land prices within metropolitan areas is crucial for identifying regional development disparities. It holds significant practical value for guiding the rational allocation of resources, optimizing land use efficiency, and promoting collaborative development across the metropolitan region. Based on the residential land auction and sale data of 48 counties in the Wuhan metropolitan area, this paper analyzes the spatial and temporal evolution characteristics and network structure of regional residential land prices in 2015, 2018, and 2021 using spatial autocorrelation and social network analysis. Further, it analyzes the factors that influence residential land prices using the MGWR model. It is found that: (1) the residential land price in the Wuhan metropolitan area shows a circle characteristic of decreasing from Wuhan as the core to the periphery, with obvious polarization characteristics, and relatively relieved in 2021. Similar aggregation types exhibit a distinct cluster distribution in space. (2) The network structure of residential land prices in the Wuhan metropolitan area increases yearly, but the evolution speed is slow. (3) Compared to OLS and GWR, the MGWR model more accurately measures the impact and spatial variability of variables on residential land prices. The contributing factors, ranked by their influence, are: shopping malls > secondary roads > population > plot ratio > parks and squares > medical facilities > GDP > entertainment venues. With the exception of population and entertainment venues, all other factors exert a positive influence on residential land prices to varying extents. Resource sharing and city-specific policies are feasible ways to promote the healthy and stable development of the land market in the Wuhan metropolitan area.

## 1. Introduction

As a significant driver of regional economic growth, metropolitan areas play a pivotal role in fostering regional synergistic development [1]. During the 14th Five-Year Plan period, they represent the primary arena for advancing China’s new urbanization strategy. The incorporation of the metropolitan area has led to an acceleration in the flow of production factors, including material, labor, information, capital, and knowledge, between cities [2]. The interaction of socio-economic activities between regions has become more complex and closer. The spatial structure of the metropolitan area has gradually evolved from uncentered polarization and decentralized agglomeration to the networked mode. The interconnected nature of this structure is evident in the transportation, population, information, and various other facets of the metropolitan area [3], as well as in the spatial distribution of urban land prices within the region [4].

The price of land is a reflection of the dynamics of supply and demand within the land market and thus serves as a crucial tool for government allocation of various types of land [5]. Among these, residential land prices play a pivotal role in guiding the operations of the real estate market and have a significant impact on residents’ fundamental living standards and societal well-being [6]. Given the close relationship between residential land prices and several factors, including location, natural ecology, urbanization level, infrastructure, public service facilities construction, and urban planning, it is to be expected that there will be discernible variations in residential land prices across different spatial locations within urban areas [7]. The accelerated integration of urban and rural development, coupled with the continuous expansion of urban space, has led to an enhanced correlation between land prices in neighboring cities [8]. It is therefore necessary to analyze the characteristics and formation mechanism of regional land prices in greater depth, from two dimensions: time evolution and spatial interaction, to meet the requirements of regional coordinated development. It is also necessary to enrich research related to metropolitan network structure to provide theoretical support and suggestions for optimizing land resource allocation, promoting metropolitan same-city development within city clusters, and facilitating factor flow between urban and rural areas.

At present, research on residential land prices is predominantly concentrated on the spatial characteristics and influencing factors of land prices within urban areas [9]. Scholars have developed theoretical analysis frameworks and mathematical models for examining the factors influencing land prices at various scales [10]. Prior research has demonstrated that macro-level drivers of land prices include social and economic development [11], regional development and construction [12], as well as urban planning adjustments [13,14]. Geographical location [15,16], neighborhood conditions [17,18], and urban road networks have been identified as crucial micro-level factors affecting land prices [19,20]. Moreover, some scholars have examined the relationship between residential land prices and specific features, including sea views, lake views, proximity to large parks, and proximity to emerging infrastructure [21]. In light of the relatively recent development of China’s land market, certain Chinese studies focus on quantifying the extent of discrimination of influencing factors and optimizing models [22]. Existing studies have primarily focused on the number of public facilities near the sample site or the straight-line distance from residences to these facilities, often overlooking the influence of factors such as topography, natural features, transportation infrastructure, and building density. As a result, there is a critical need to incorporate these elements when assessing the spatial accessibility of public service facilities. Spatial accessibility not only reflects residents’ actual access to these services but also illustrates the impact of geographic location on their convenience. Commonly used methodologies in spatial accessibility measurement include the Cumulative Opportunity Model (COM), the Supply-Demand Ratio Model (SDRM), and various models derived from the Two-Step-Forward-Finding Approach (2SFCA), such as the i2SFCA [23]. In addition, spatial accessibility analysis using Rhino + UNA tools is also an effective research avenue [24].

In the field of research methodologies about residential land prices, the hedonic price model (HPM), spatial econometric model, and geographically weighted regression model have been utilized to examine the spatial correlation effects and driving factors influencing residential land prices. The latter two models exhibit a markedly superior goodness of fit compared to the former [25]. While the HPM primarily addresses the linear regression (OLS) relationship between housing prices and associated factors, it lacks an exploration of the spatial relationships between these elements [26].The GWR model is capable of capturing the spatial characteristics of land prices and elucidating the mechanisms through which various influencing factors impact land prices, thus garnering attention from the academic community [27]. Furthermore, GWR delves into the spatial heterogeneity between housing prices and influencing factors beyond traditional regression methods, addressing the limitations of HPM [28]. Spatial econometric models are primarily concerned with examining spatial correlation and heterogeneity among economic phenomena, particularly in elucidating the mechanisms through which related factors impact land price correlations [29]. GWR assumes that the regression coefficients for each location are determined by a fixed bandwidth. However, spatial data often exhibit varying densities and distributions, which a fixed bandwidth may fail to capture, potentially impairing model performance. In contrast, Multi-Scale Geographically Weighted Regression (MGWR) allows each explanatory variable to have its own bandwidth, enabling the capture of local spatial heterogeneity across multiple scales. This improves both prediction accuracy and model robustness [30]. Thus, MGWR provides significant advantages in addressing bandwidth selection and enhancing model performance. Additionally, some researchers combine Geographically Weighted (GW) models with artificial intelligence (AI) techniques [31], offering innovative approaches.

The majority of research on land prices is currently concentrated on individual cities [32,33], with a relatively limited investigation into the spatial network structure of land prices that integrates regional urban agglomerations and specific demands for coordinated development [34]. The Wuhan metropolitan area plays a pivotal role in the urban agglomeration of the Yangtze River Basin in China [35]. It is a pivotal region for implementing the strategy of central China’s development, deepening comprehensive reforms, and promoting new urbanization [36]. In recent years, the coordinated development process of the Wuhan metropolitan area has experienced a notable acceleration, which has given rise to more sophisticated demands for optimizing regional land resource allocation, guiding factor flow, and developing spatially new urban industrial areas [37]. This progress underscores the significance of strategic planning and efficient resource management in propelling sustainable growth within this pivotal region. The Wuhan metropolitan area has currently formed a multi-dimensional intercity network structure, with Wuhan and Ezhou as the main and sub-centers, and Huangshi-Xiaogan-Huanggang as agglomeration-radiation zones. Wuhan exerts a dominant influence in terms of land market attractiveness, economic development, and real estate demand. In contrast, neighboring cities exhibit lower levels of land market activity, characterized by limited transaction volumes and substantially lower total transaction values. This regional disparity in the land market is likely to exacerbate the development gap between the central city and its surrounding areas, resulting in an overconcentration of resources in Wuhan while restricting the growth potential of peripheral cities. Furthermore, such market imbalances may impede the overall sustainable development of the metropolitan area, diminishing its competitive edge in both national and global contexts.

The majority of extant studies have primarily investigated the driving forces behind the spatial differentiation of residential land prices in urban areas from a static standpoint [38]. However, there is a paucity of studies that adopt a “flow” perspective to describe the shaping of regional land price network spatial structure by the interaction between production factors and city interface [39,40]. With the accelerated flow of information, resources, and capital, land prices have evolved from a simple regional issue to a phenomenon exhibiting network characteristics across a broader spatial scale. The strengthening interconnections among cities within the Wuhan metropolitan area have led to increasingly networked and nonlinear dynamics in land price variations. Therefore, social network analysis provides a rational approach to understanding this complex phenomenon. Through social network analysis, the interrelationships of land prices between different cities or regions can be revealed, enabling a deeper exploration of the propagation mechanisms and interaction patterns of land prices within the metropolitan area’s internal network structure. To date, only a limited number of scholars have employed the social network analysis approach to investigate the network structure characteristics of the house price correlations among cities [41,42]. Nevertheless, all of these studies have demonstrated the applicability of the social network analysis method in the context of the land price market, providing both data support and theoretical foundations for formulating targeted regulatory policies for different cities. Therefore, it is crucial to address whether the Wuhan metropolitan area has developed a network structure for residential land prices and how to coordinate the competition in the residential land markets among cities within the region.

This paper takes 48 counties in the Wuhan metropolitan area as the research object, based on the residential land grant land price data, urban point of interest (POI), OSM road network, and other data in 2015, 2018, and 2021, analyzes the regional correlation effect of residential land price by using spatial autocorrelation method, and explores the network structure characteristics of residential land price in the metropolitan area by using social network analysis method. Finally, the geographically weighted regression model is used to explore the influencing factors and driving mechanisms of the spatial characteristics of land prices in the overall region. This study is devoted to addressing the following three inquiries: Does the Wuhan metropolitan area have a network structure of land prices? What are the characteristics of the land price network structure in the Wuhan metropolitan area? What are the factors and mechanisms influencing the formation of land price spatial structure characteristics? The responses to the aforementioned inquiries provide a theoretical foundation and empirical evidence that inform improvements to land market regulation within the Wuhan metropolitan area and facilitate the acceleration of synergistic regional development.

## 2. Materials and methods

### 2.1. Research area

**Fig 1:**
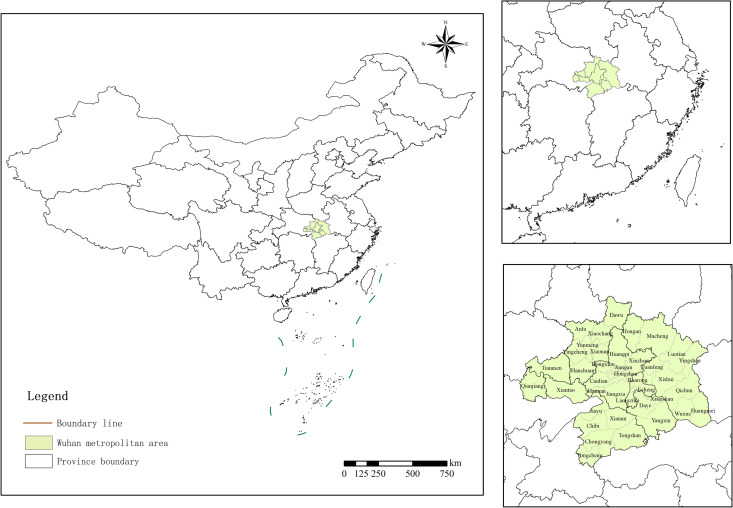
Research area in Hubei Province, China.

The Wuhan metropolitan area also referred to as the “8+1” metropolitan area of Wuhan, encompasses nine regions: Wuhan, Huangshi, Ezhou, Huanggang, Xiaogan, Xianning, Xiantao, Qianjiang, and Tianmen (Fig 1). It spans 48 counties (cities and districts) and is strategically positioned at the crossroads of China’s two primary development axes from east to west and north to south. Occupying a central position among the five provinces in the central region. With a land area of 58,051 square kilometers accounting for approximately 31% of Hubei province’s total area; in 2021, the Wuhan metropolitan area housed around 54% of the province’s permanent population and contributed to about 60% of its GDP. The industrial composition was as follows: 7.08% from the primary sector, 37.01% from the secondary sector, and 55.91% from the tertiary sector.

### 2.2. Data and pre-processing

#### 2.2.1. Fundamental data collection.

**Fig 2:**
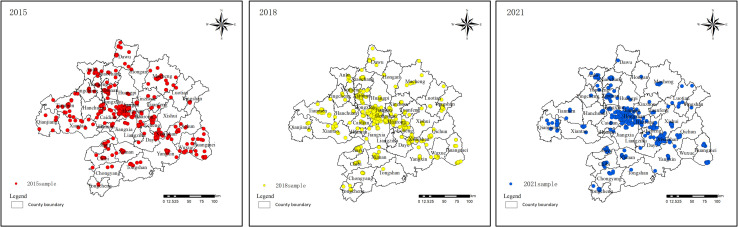
The sample in 2015, 2018, and 2021.

The study primarily utilized data from four key aspects:

1. Residential land price data. The data were obtained from the China Land Market Network (https://www.landchina.com), which provides land price information with the advantages of temporal continuity, a large sample size, and comprehensive data. As the primary method of paid land transfer in China is the auction, and as this method provides a more accurate reflection of the status of land resource supply and demand allocation. From 2015 to 2021, the integration of the Wuhan metropolitan area yielded initial results, representing a critical phase in the steady promotion of urban integration. The years 2015, 2018, and 2021 correspond to key stages in the process: initiation, deepening, and the emergence of early outcomes. A comparative analysis of these three time points reveals the impact of policy shifts, infrastructure development, and regional economic integration on residential land price changes, offering valuable insights into the long-term effects of the Wuhan metropolitan area’s integration on the land market. Therefore, this paper selects the residential land auction data of 48 counties in the Wuhan metropolitan area in 2015, 2018, and 2021. The dataset includes basic information such as the name, transaction price, address, land area, land use, usage duration, floor area ratio, and land user rights holder. The land price per unit area is calculated based on the ratio of transaction price to land area. Using the Geocoding service provided by the Baidu Open Platform (https://lbsyun.baidu.com/), an API interface is applied to convert the land parcel address data into corresponding coordinate points, enabling the spatial localization of land transfer parcels. According to China’s Civil Code, the usage rights for residential land, which are granted for a 70-year term, may be automatically renewed upon expiration. Therefore, the remaining duration of state-owned land use rights does not impact current land prices. The floor area ratio of the parcels will be considered as an influencing factor in subsequent analyses. Parcels with severe data deficiencies account for less than 1% of the total sample size. Due to their negligible proportion, these few parcels with missing data will be excluded from the analysis. Ultimately, the final dataset includes 506, 568, and 487 valid land price samples, each containing spatial geographic information (Fig 2). The normality of the land price sample points was assessed using the Kolmogorov-Smirnov (K-S) test after the log was transformed. A p-value of less than 0.01 indicated a significant normal distribution, thereby meeting this study’s requirements.

1. Map and road network data. The administrative division data is sourced from the open platform (https://github.com/ruiduobao/shengshixian.com). The road network vector data for the study area were obtained from OpenStreetMap (https://m.osmtools.de). ArcGIS was used to extract four types of roads—highways, main roads, secondary roads, and tertiary roads—forming the road network [43]. The road network has been topologically checked with no overlapping points, no pseudo-nodes, and no intersections.
2. GDP and population spatial distribution kilometer grid dataset. The data were obtained from the Resource Environment Science Data Platform (https://www.resdc.cn). The data are presented in the form of a 1 km × 1 km grid, with each grid representing the total GDP and population within the specified area. This reflects the detailed spatial distribution of GDP and population within the study region. The GDP and population within the grid cells of the monitoring points are quantified and used for subsequent analysis in the MGWR model.
3. POI data. The data pertaining to points of interest (POIs) of public transportation, medical facilities, parks and squares, shopping malls, schools, and entertainment venues were obtained from the Resource and Environmental Science Data Platform (https://www.resdc.cn/data.aspx?DATAID=178).
4. The resident population data. The Hubei Provincial Statistical Yearbook has collected the data of the resident population in each county, which is used to modify the gravity model.

#### 2.2.2. Influencing factors.

The residential land price at the monitoring sample points is used as the dependent variable. Based on urban spatial organization theories such as supply and demand theory, sector theory, multi-core theory, neighborhood unit theory, and community life circle theory, ten influencing factors related to transportation, neighborhood, and economics are selected as independent variables (Table 1). The aim is to analyze the impact of these factors on the spatial characteristics of residential land prices in the Wuhan metropolitan Area.

**Table 1:** Selection and description of influencing factors.

Type	Influencing factors	Description of influencing factors	VIF
Traffic factors	Public transportation	The number of arriving bus and subways within 800m of the road network of the monitoring point	1.57
	Secondary roads	The number of arriving secondary roads within 800m of the road network of the monitoring point	1.82
Neighborhood factors	Medical facilities	The number of arriving medical facilities within 800m of the road network of the monitoring point	3.86
	Parks and squares	The number of arriving parks and squares within 800m of the road network of the monitoring point	1.32
	Shopping malls	The number of arriving shopping malls within 800m of the road network of the monitoring point	1.14
	Schools	The number of arriving primary and secondary schools within 800m of the road network of the monitoring point	1.64
	Entertainment venues	The number of arriving entertainment venues within 800m of the road network of the monitoring point	3.02
Economic factors	GDP	The GDP within the 1 km*1 km grid containing the monitoring point.	2.37
	Population	The population within the 1 km*1 km grid containing the monitoring point.	2.67
Other Factors	Plot ratio	The floor area ratio of the monitoring point	1.38

The “Urban Residential Area Planning and Design Standards (GB50180-2018)” serves as a key guideline for the planning and construction of residential areas and surrounding facilities in China. The accessibility of infrastructure and public services around residential areas largely determines land price levels. In selecting transportation-related factors, the accessibility of monitoring sample points to public transportation and secondary roads is primarily considered. For neighborhood-related factors, the focus is on the accessibility of medical facilities, parks and square, shopping malls, primary and secondary schools, and entertainment venues. A detailed description of the indicators is provided in Table 1. This study mainly employs the Urban Network Analysis (UNA) tool in Rhino to assess the accessibility of these factors.

In accordance with the “Community Life Circle Planning Technical Guidelines (TD/T 1062-2021),” the 15-minute community life circle is defined as the spatial range that meets the basic living needs of residents [44]. It also serves as a key indicator for assessing the value of land parcels. During the UNA analysis, residential sample points are treated as the origin, with service facilities as the destination. Roads are abstracted into a network, and the 15-minute community life circle (800 meters) is used as the coverage area for daily services. The analysis quantifies the number of public transportation options, secondary roads, medical facilities, parks and square, shopping malls, primary and secondary schools, and entertainment venues within this 800-meter radius.

Economic factors, including GDP and population, influence land prices through their effects on market demand and supply. To better capture the spatial distribution of GDP and population, this study uses 1 km × 1 km grid data. The floor area ratio, which directly impacts land development potential and market demand, is also considered. The plot ratio at each residential sample point is extracted from the base land price data as one of the key factors.

This article uses the variance inflation factor (VIF) to detect multicollinearity among model variables in order to ensure the credibility of the model estimation results. The test outcomes demonstrate that the VIF coefficients of the variables are all less than 10, indicating the absence of multicollinearity concerns.

### 2.3. Methods

#### 2.3.1. Spatial autocorrelation.

Spatial autocorrelation is commonly used to measure the existence of spatial agglomeration of residential land prices [45,46]. In this study, the Moran’s I index is employed to elucidate the prevailing characteristics of the spatial correlation of residential land prices at the county level. The model is expressed in equation (1).


$$I=\frac{n\sum_{i=1}^{n}\sum_{j=1}^{n}W_{ij}\left(x_{i}-\overset{―}{x}\right)\left(x_{j}-\overset{―}{x}\right)}{\sum_{i=1}^{n}\sum_{j=1}^{n}W_{ij}\sum_{i=1}^{n}{\left(x_{i}-\overset{―}{x}\right)}^{2}}\;$$ (1)

where xi and xj are the observed values of residential land prices in county-level cities i and j, respectively, n is the number of counties, is the average value of observations, and Wij is the spatial weight matrix for adjacency. Moran’s I index ranges from -1 to 1, Moran’s I > 0 indicates a positive spatial correlation and Moran’s I < 0 indicates a negative spatial correlation, the absolute value of Moran’s I index reflects the degree of spatial correlation, the larger the absolute value, the stronger the degree of spatial correlation, and vice versa. Moran’s I index absolute values reflect the degree of spatial correlation, larger absolute values reflect the degree of spatial correlation, and smaller absolute values reflect the degree of spatial correlation.

Global spatial autocorrelation can reveal the overall spatial distribution of observations, but it is difficult to detect the location of agglomerations and the degree of regional correlation, and it cannot indicate the structure and characteristics of agglomerations among prefectures. Local spatial autocorrelation (LISA) is used to reveal the degree of correlation between each spatial unit and its neighboring units with respect to a certain attribute, which can further reveal the location and extent of spatial agglomeration or disagglomeration [47]. The relationship between Moran’s index of the evaluation unit and the average value of Moran’s index of its neighboring units allows for the classification of these units into four types: HH (high-high), LL (low-low), HL (high-low), LH (low-high), and LH (low-high), which are modeled as shown in equation (2).


$$I_{i}=\frac{n^{2}\left(x_{i}-\overset{―}{x}\right)\sum_{j=1}^{n}W_{ij}\left(x_{j}-\overset{―}{x}\right)}{\sum_{i=1}^{n}\sum_{j=1}^{n}W_{ij}\sum_{j=1}^{n}\left(x_{j}-\overset{―}{x}\right)}$$ (2)

where Ii is the value of local spatial autocorrelation index, xi、xj、n and Wij have the same meanings as in equation (1). A positive Ii indicates that residential land values in neighboring counties are similar (HH or LL) and a negative Ii indicates that residential land values in neighboring counties are not similar (HL or LH).

#### 2.3.2. Social network analysis.

This study establishes a residential land price linkage network system through the measurement of the strength of land price linkages among counties. The observation of both overall and local network characteristics allows for the identification of the roles and positions of residential land prices in counties within the Wuhan metropolitan area within the network structure and degree of connectivity [48].

1. Gravity models represent a significant methodology for measuring the strength of inter-city connections, forming the basis for social network analyses by establishing a matrix of residential land prices [40]. The traditional gravity model has some defects: the Euclidean distance only characterizes the physical distance in space, which cannot reflect the actual cost of access [49]. In order to show the basic pattern of urban spatial connectivity more deeply, this paper corrects the distance D between cities. Using the ArcGIS cost-distance analysis tool, the spatial distance is transformed into the temporal distance. To obtain the minimum travel time Dij between nodes i and j, i, the temporal distance cost of access between districts, the area is rasterized and appropriate speed and time cost values are assigned to different levels of traffic routes (Table 2).

**Table 2:** Speed settings for various modes of transportation.

Mode of transportation	High speed access	Grade one road	Secondary road	Tertiary roads
Set of speed (km/h)	100	80	60	30

The calculation formula is as follows:


$$K_{ij}=\frac{\sqrt{P_{i}M_{i}}\sqrt{P_{j}M_{j}}}{D_{ij}}$$ (3)

where Kij is the strength of association between two counties, Pi、Pj are the resident population of the counties, Mi、Mj are the average land prices of residential sample points within the counties, Dij is the time distance between the two counties.

1. Social network analysis allows for a reasonable interpretation of the characteristics of the relationships between counties in the land price network. The land price association strength matrix obtained from the gravity model is binarized, and the overall and local network structure is analyzed with the support of Ucinet [50].

Network density is employed to characterize the overall network linkage status and to quantify the spatial correlation of residential land prices among counties.


$$D=\frac{2L}{N(N-1)}$$ (4)

where D s the value of network density; The number of pairs of points in the network that have an actual existing relationship is denoted as L; The number of counties is N.

The measurement of individual network characteristics is primarily conducted through the assessment of three key centrality metrics: degree centrality, betweenness centrality, and closeness centrality [51]. Degree centrality measures the number of paths in a network that connect a given individual directly to other individuals, betweenness centrality portrays the sum of the distances of shortcuts constructed by a given member to other members, and closeness centrality measures the role of a given member as a “bridge” in the structure of the network.

#### 2.3.3. Multi-Scale Geographically Weighted Regression (MGWR).

The GWR model can better reflect the impact of variables under differentiated spatial characteristics than traditional econometric models [52]. This is because it considers the influence of spatial autocorrelation and heterogeneity on the model, making it a more scientific approach. The specific formula is as follows:


$$\begin{array}{c} y_{i}=\beta \left(u_{i},v_{i}\right)+\sum {\mathrm{\backslash nolimits}}_{k=1}^{n}\beta_{k}\left(u_{i},v_{i}\right)x_{ik}+\varepsilon_{i} \end{array}$$ (5)

where: yi represents the residential land price at the i-th monitoring point, (ui, vi) is the projected coordinates of the i-th monitoring point, the regression coefficient βk(ui, vi) represents the k-th explanatory variable’s coefficient at i, n is the number of explanatory variables, xik is the value of the kth explanatory variable at monitoring point i, and εi is the error term.

The optimal bandwidth can be found by minimizing some model fitting goodness-of-fit diagnostics [53], which can be selected through cross validation (CV) or the Akaike information criterion (AIC) width value. The formula is as follows:


$$\begin{array}{c} CV(b)=\sum {\mathrm{\backslash nolimits}}_{i=1}^{n}{\left[y_{i}-{\hat{y}}_{\neq i}(b)\right]}^{2} \end{array}$$ (6)

The accuracy of the GWR model depends on the choice of bandwidth, as different bandwidths often exhibit distinct spatial effects. A fixed bandwidth may fail to capture such spatial variability [54]. Therefore, this study employs the Multi-Scale Geographically Weighted Regression (MGWR) model, which automatically optimizes the bandwidth for each variable. This approach reduces the subjectivity associated with bandwidth selection in traditional GWR and allows for the identification of the varying influence of different variables across multiple spatial scales. The specific formula is as follows:


$$\begin{array}{c} y_{i}=\beta_{bw0}\left(u_{i},v_{i}\right)+\sum {\mathrm{\backslash nolimits}}_{k=1}^{n}\beta_{bwk}\left(u_{i},v_{i}\right)x_{ik}+\varepsilon_{i} \end{array}$$ (7)

where: the other parameters are the same as in GWR equation (5) and bw represents the bandwidth used for the regression coefficients of the different variables.

## 3. Results

### 3.1. Spatial distribution of residential land prices

**Fig 3:**
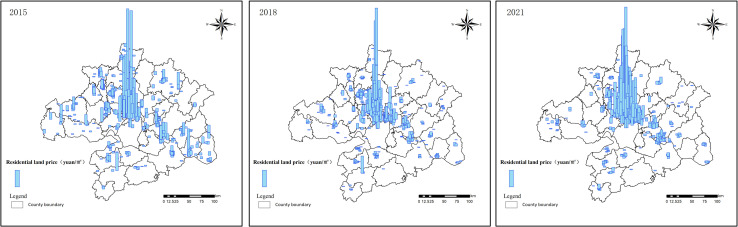
Spatial distribution of residential land prices in 2015, 2018, and 2021.

Descriptive statistical analysis of land price data in Wuhan metropolitan area (Table 3). From 2015 to 2021, the average land price increases from 2,469.4 to 7,368.8, nearly tripling, with the land market in the Wuhan metropolitan area developing better. The median land price increases from 1,347.9 to 2,595, a faster growth rate, but still far below the average value, indicating that land prices in a few high-value areas (such as the urban core areas) are skyrocketing, driving up the overall average value. A comparison of the data reveals that by 2018, this polarization effect had intensified in comparison to 2015, resulting in significant disparities in land price levels across different regions (Fig 3). However, by 2021, this phenomenon had been mitigated, resulting in a gradual reduction in the disparity of residential land prices in surrounding areas and ultimately achieving a preliminary balance in development within the metropolitan area.

**Table 3:** The statistics of residential land price.

	Mean (yuan/m²)	Maximum value (yuan/m²)	Upper quartile (yuan/m²)	Median (yuan/m²)	Ower quartile (yuan/m²)	Minimum value (yuan/m²)	Standard deviation	Kurtosis
2015	2469.4	44365	2400.3	1347.9	726.1	43.7	3980.8	53.4
2018	4717.8	130760	5090.1	2056.2	1059.2	229.4	9567	93.3
2021	7368.8	122220	6155	2595	1455.2	425	14398	24.4

### 3.2. Spatial agglomeration characteristics of residential land price

The Moran’s I values for the Wuhan metropolitan area in 2015, 2018, and 2021 were 0.246, 0.262, and 0.144, respectively. These values indicate a positive spatial correlation in residential land prices, meaning that areas with high residential land prices are adjacent to each other. The p-values were all below 1% through significance testing, and the z-values were all greater than 1.65, indicating a significant spatial clustering. For a detailed overview of the results, please refer to Table 4.

**Table 4:** Global spatial autocorrelation statistics results.

Year	2015	2018	2022
I value	0.2464	0.2623	0.1436
Z value	5.1856	6.3093	3.4482
P value	0.0000	0.0000	0.0005

**Fig 4:**
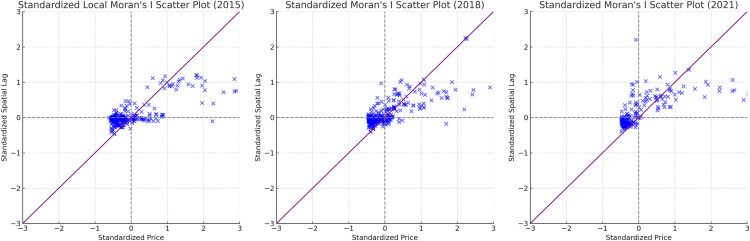
LISA scatterplot of residential land price in 2015, 2018 and 2021.

The localized Moran’s index is measured by Eq. (2) and the results are shown in Fig 4. The LISA scatter plot reveals a clear positive correlation, primarily distributed in the first and third quadrants. In 2015, the scatter points are relatively concentrated, but as time progresses, the distribution area gradually expands, suggesting that high land price regions may no longer consistently align with neighboring areas of high land prices.

**Fig 5:**
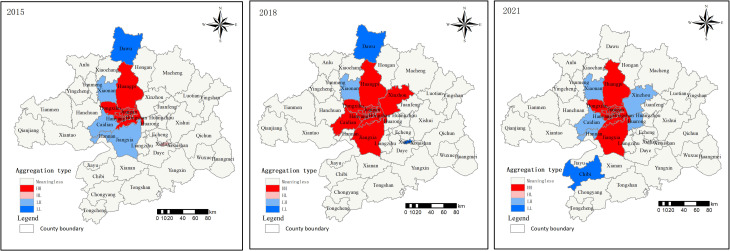
LISA cluster of residential land price in 2015, 2018 and 2021.

The aggregation patterns across different counties are depicted in Fig 5. In 2015, a total of nine cluster areas were identified, which are mainly distributed in the central urban areas of Wuhan, such as Jiangan, Qiaokou, and Huangpi. By 2018, the number of HH cluster areas had increased to 12, with the inclusion of Xinzhou, Jiangxia, and Caidian. However, by 2021, there was a reduction in the extent of HH cluster areas as Xinzhou and Caidian transitioned into LH cluster types. The LL cluster type was initially observed in Dawu in both 2015 and 2018 but shifted to Chibi by 2021. Furthermore, from 2015 to 2018, there was a transformation from the HL cluster type to the LL cluster type within Xialu. The findings indicate a significant spatial correlation among residential land prices within the Wuhan metropolitan area, with discernible clustering patterns for similar types. It also reflects the mono-core polarization of the Wuhan metropolitan area.

### 3.3. Spatial network characteristics of residential land price

#### 3.3.1. Overall network structure.

The application of social network analysis revealed that the density values of the residential land price network in the Wuhan metropolitan area were 0.3626, 0.3954, and 0.4051 in 2015, 2018, and 2021, respectively. This observation indicates the presence of a distinctive network characteristic within the residential land prices of the Wuhan metropolitan area. From 2015 to 2018, network density showed a slight increase of 9%, indicating a strengthened land price connection between counties within the Wuhan metropolitan Area. This can be closely linked to a series of initiatives under the comprehensive reform pilot of the “Two-Oriented” Society construction during this period. However, from 2018 to 2021, the growth rate of network density slowed to 2.5%. This trend aligns with the outbreak of the COVID-19 pandemic in 2020, which caused widespread business shutdowns, decreased consumer demand, and reduced investment, thereby severely impacting economic development and slowing socio-economic exchanges across regions.

#### 3.3.2. Individual network structure.

The influence of several driving factors has resulted in the emergence of notable regional disparities in the “position” of land within the land price network. Subsequently, an in-depth examination was undertaken of the degree centrality, closeness centrality, and betweenness centrality of the land price network within the Wuhan metropolitan area.

**Fig 6:**
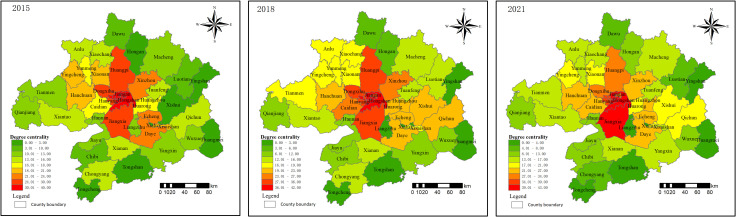
Spatial distribution of degree of land price network in 2015, 2018 and 2021.

**Fig 7:**
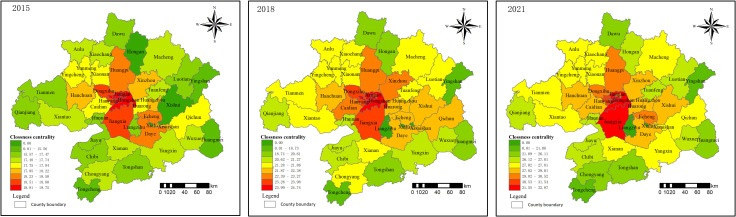
Spatial distribution of closeness of land price network in 2015, 2018 and 2021.

**Fig 8:**
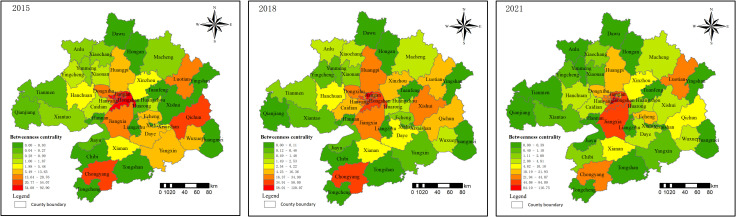
Spatial distribution of betweenness of land price network in 2015, 2018 and 2021.

1. The degree of centrality is a measure of the level of urban agglomeration land prices in the core position of the network structure. As illustrated in Fig 6, the urban agglomeration land price network is currently dominated by a small number of nodes, with notable disparities in the competitiveness of counties. The areas occupying the core position in the network are Hongshan, Wuchang, and Jiangan, with degree centrality values exceeding 40 in 2015, 2018, and 2021, respectively. Areas such as Echeng, Daye, Hanchuan, and Xishui exhibit relatively high competitiveness, as indicated by degree centrality values exceeding 20, and have become relatively central regions within the network. Nodes such as Tongshan, Tongcheng, Huangmei, and Yingshan exhibit degree centralities below 5, indicating their peripheral position within the network. In summary, while there are only a limited number of high-value agglomeration areas in the surrounding regions, these areas have developed effectively to form a stable hinterland layer. From a temporal perspective, analysis indicates minimal fluctuation in degree centralities among the 48 regions, reflecting a stable development of residential land price networking within the Wuhan metropolitan area. This indicates that an increasing number of counties are engaging in the coordinated development of regional residential land prices.

1. Closeness centrality is used to analyze the convenience of residential land prices being connected to other areas. In 2015, the maximum closeness centrality was 19.75 in Hongshan and the minimum was 15.31 in Tongcheng; in 2018, the maximum closeness centrality was 24.74 in Jiangan and the minimum was 17.94 in Tongcheng; in 2021, the maximum closeness centrality was 32.87 in Hongshan and the minimum was 20.98 in Tongcheng. The relatively large distance of the road network from Tongcheng County to other counties may result in less convenient external transportation, leading to the lowest degree of centrality. Over time, the centrality of most areas has shown improvement (Fig 7), and inter-regional convenience has gradually increased, giving rise to a step-by-step formation of the Wuhan metropolitan area land price network.

1. The betweenness centrality measures the “bridge” role of nodes in the network structure. In 2015, the overall betweenness centrality was 7.56%, increasing to 10.17% in 2018, and then slightly decreasing to 9.74% in 2021. Overall (Fig 8), the Wuhan metropolitan area has improved the accessibility and stability of the network. The central urban area of Wuhan still maintains a leading position in terms of betweenness centrality, with the most significant increase seen in Luotian, which rose from 16.74 in 2015 to 44.07 in 2021. Luotian is developing the Dabie Mountain Hundred-Mile Yangtze River Ecological Corridor and implementing the “Tourism-Driven County” policy. The development of the tourism industry has stimulated land price growth, positioning the county as a “bridge” within the network structure.

### 3.4. Influencing factors on residential land

#### 3.4.1. Model solution.

The previous section compared three time points—2015, 2018, and 2021—to reveal the spatiotemporal characteristics of residential land price changes during the integration process. By 2021, the Wuhan Metropolitan Area had achieved initial results, reflecting the cumulative effects of long-term policy implementation and urban development planning. Focusing on this year for analysis provides a more comprehensive view of the combined impact of various influencing factors on residential land prices. Therefore, this study focuses on the residential land prices in 2021, further analyzing the factors influencing the spatial differentiation of land prices and the underlying driving mechanisms. In the MGWR model construction process, an adaptive Gaussian function is selected, and the AICc criterion is used for optimization. Variables are standardized during the analysis. To assess the superiority of the MGWR model, the results of the OLS model, GWR model, and MGWR model are compared, with the detailed findings presented in Table 5.

**Table 5:** Comparison of fitting results between OLS and GWR.

Model	R^2^	Adjust R^2^	AIC	AICc	BIC
OLS	0.524	0.514	1042.695	1045.353	–
GWR	0.814	0.781	709.658	736.059	1016.699
MGWR	0.825	0.804	640.421	653.650	862.473

The Adjusted R^2^ of the MGWR model is 0.804, which is higher than that of the OLS and GWR models, indicating that the MGWR model has greater explanatory power and can account for 80.4% of the variation in residential prices within the Wuhan metropolitan Area. Additionally, the AICc values of the MGWR model are lower than those of the OLS and GWR models, suggesting that the MGWR model provides more accurate results with fewer parameters, thus offering a better fit.

To assess the statistical significance of the influencing factors, local t-values are compared with the Adjusted t-value (95%). In the MGWR regression results, the coefficients for eight factors—GDP, secondary roads, medical facilities, parks and squares, shopping malls, entertainment facilities, plot ratio, and population —are statistically significant, while the coefficients for public transportation and schools are not. These factors show generally low local t-values and are therefore excluded from further analysis.

Compared to the fixed bandwidth of 146 in the GWR model, the MGWR model computes bandwidths ranging from 84 to 485 for different variables, indicating spatial heterogeneity in the impact of each variable on residential prices, with varying degrees of influence. The bandwidths for GDP, entertainment facilities, and medical venues all exceed 350, suggesting low spatial heterogeneity and a broader, influence on residential prices. In contrast, the bandwidths for parks and squares, shopping malls, plot ratio, and population range between 100 and 200, indicating moderate spatial heterogeneity and a significant local impact on residential prices. The bandwidth for secondary roads is 84, reflecting higher spatial heterogeneity and a notable impact on residential prices within smaller areas, with considerable variation across regions.

The model’s regression coefficients are presented in Table 6. Based on the absolute values of the standard deviations, the factors are ranked in terms of their contribution as follows: shopping malls > secondary roads > population > plot ratio > parks and squares > medical venues > GDP > entertainment facilities. Further visualization analysis is conducted using ArcGIS tools.

**Table 6:** MGWR model estimation results of residential land price changes.

Variable	Bandwidths	Confidence				
		Mean	Std	Min	Median	Max
(Intercept)	88.000	−0.046	0.431	−0.470	−0.243	0.869
Public transportation	485.000	0.014	0.003	0.009	0.015	0.020
Secondary roads	84.000	0.097	0.213	−0.144	−0.001	0.517
Medical venues	380.000	0.204	0.024	0.159	0.216	0.231
Parks and squares	172.000	0.072	0.096	−0.046	0.058	0.243
Shopping malls	132.000	0.143	0.262	−0.068	0.012	0.687
Schools	485.000	−0.034	0.001	−0.036	−0.034	−0.031
Entertainment facilities	485.000	−0.154	0.002	−0.159	−0.155	−0.146
GDP	380.000	0.172	0.008	0.151	0.172	0.184
Population	202.000	−0.197	0.099	−0.320	−0.213	−0.062
Plot ratio	251.000	0.187	0.098	0.018	0.207	0.292

#### 3.4.2. Intercept.

**Fig 9:**
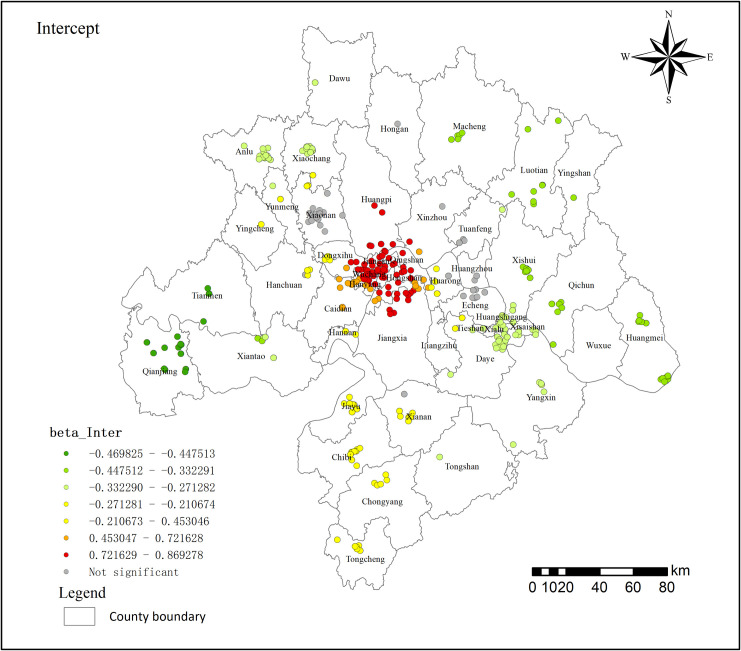
Intercept regression coefficient distribution in 2021.

The intercept term represents the baseline level of the Wuhan metropolitan area, indicating the initial value of residential land prices in the absence of spatial heterogeneity or other influencing variables. Spatially (Fig 9), the variation of the intercept term exhibits clear spatial heterogeneity, with residential land prices following a concentric pattern that decreases from the central urban area of Wuhan outward. The residential land prices display a concentric pattern, with decreasing values extending from the central urban region of Wuhan towards its outer periphery. The first concentric zone comprises Jiangan, Jianghan, and Wuchang, which are distinguished by the highest land prices. The second concentric zone encompasses Huangpi District, Jiangxi, and Xial. The third concentric zone includes Hanchuan, Hongan, and Huaron. Luotian and Tongshan exhibit the lowest land prices. Although there is a discernible continuity in land prices across the region, there is also a notable degree of spatial heterogeneity. As the spatial decrease towards the periphery progresses, Chongyang County and Qianjiang exhibit alternating high- and low-value anomalies. The considerable difference in intercept values between the central and peripheral regions may contribute to the phenomenon of urban “polarization,” potentially impeding the efficient allocation of resources and the balanced development of the Wuhan metropolitan area.

#### 3.4.3. Traffic factors result.

**Fig 10:**
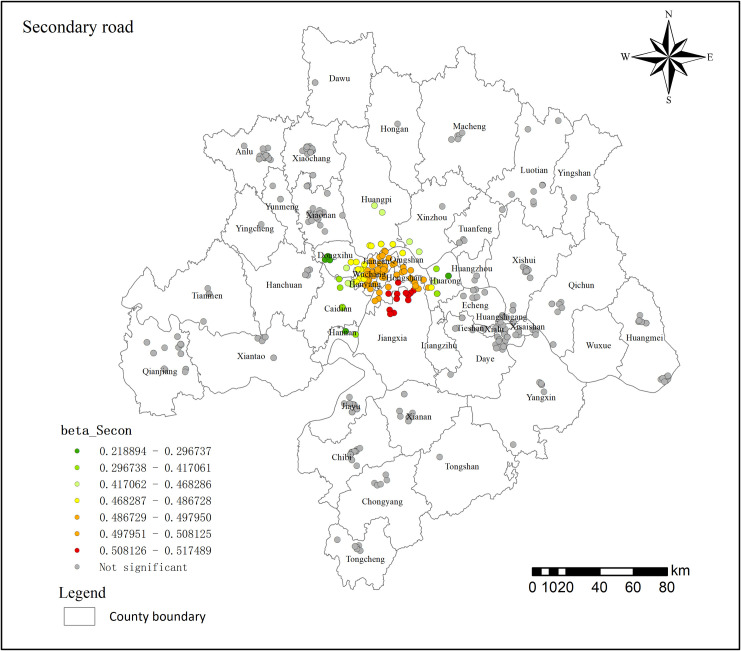
Transportation factor regression coefficient distribution in 2021.

The MGWR model results indicate that 20.02% of the outcomes passed the significance test, with regression coefficients ranging from 0.219 to 0.517 (Fig 10), exhibiting clustering around the central area of the Wuhan metropolitan region. In the significant areas, the presence of more secondary roads, higher road network density, and greater travel convenience are associated with relatively higher land prices. This phenomenon reflects the increased emphasis on accessibility by residents in suburban districts such as Jiangxia and Hongshan. The density of secondary road networks significantly enhances the attractiveness and market value of residential properties. The gray areas indicate that secondary roads do not significantly affect residential land prices in these regions, likely because residents in non-central cities within the metropolitan area have smaller travel ranges, and transportation convenience is not a primary factor in their housing decisions.

#### 3.4.4. Neighborhood factors result.

**Fig 11:**
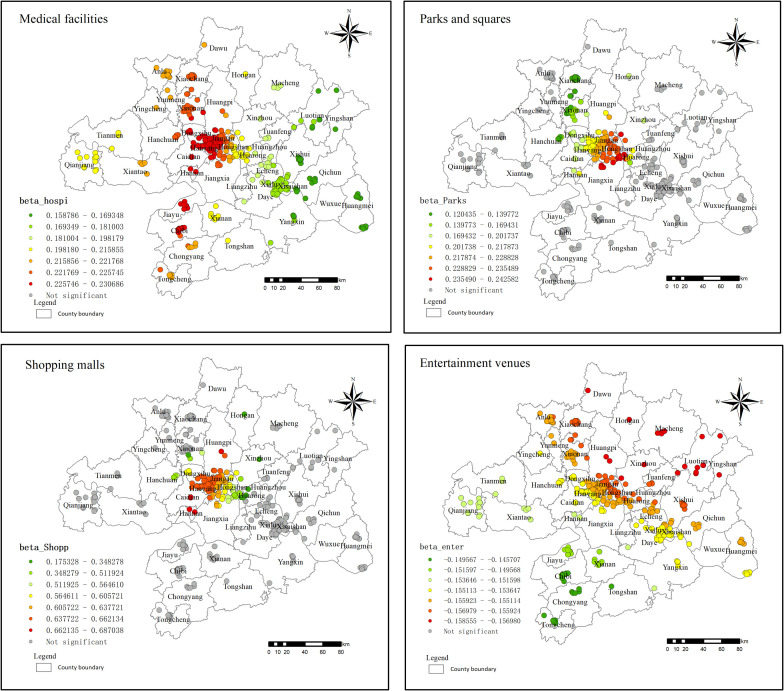
Neighborhood factor regression coefficient distribution in 2021.

The regression coefficients for medical facilities range from 0.159 to 0.231, with overall statistical significance (Fig 11). The influence of medical facilities decreases concentrically in an elliptical pattern, with the major axis roughly aligned in the north-south direction, and extending toward the northeast and southwest. The MGWR results reveal a notable spatial variation in the regression coefficients for medical facilities. The outbreak of the COVID-19 pandemic significantly increased public attention to health and medical services, and the strain on medical resources during the pandemic heightened residents’ reliance on these services. In counties such as Xiaonan, Chibi, and Jiayu, the highest regression coefficients range from 0.226 to 0.231, indicating relatively scarce medical resources in these areas. Residents in these regions are willing to pay higher prices for proximity to medical facilities, thereby driving up residential land prices.

In 2021, the regression coefficient for residential land prices in park and squares passed the significance test for 34.29% of the cases, exhibiting a positive influence. In areas such as Hanyang, Xinzhou, and Hongan, the proximity of parks and squares to residents’ living areas positively correlates with an increase in residential land prices. The parks and squares in these areas are progressively integrating to establish a network, resulting in an expanding service coverage radius and heightened influence of parks and squares on residential land prices.

The regression coefficients for significant points near large shopping malls range from 0.175 to 0.687, suggesting that residential land prices are higher in proximity to these malls. As the distance from the metropolitan center increases, the regression coefficient initially remains stable between 0.638 and 0.662. However, in districts such as Caidian, Hannan, and Huangpi, the coefficient rises to 0.687, before gradually decreasing toward the outskirts. The establishment and operation of shopping malls are typically linked to high levels of economic activity, and nearby areas often attract developers’ attention. In regions where land supply is insufficient to meet demand, residential land prices tend to be higher. Shopping malls also generate spillover effects, enhancing the commercial value and overall living standards of surrounding areas. This is particularly evident in urban expansion zones, where large shopping malls play a crucial role in driving up land prices, acting as a significant incentive for land development.

The regression coefficients for entertainment venues across all sample points in the study area are significant, ranging from −0.156 to −0.149. The small absolute values suggest that residential land prices are not highly sensitive to the presence of entertainment venues. Furthermore, as the number of entertainment venues within a 15-minute (800m) walking distance increases, residential land prices tend to decrease. A possible explanation for this is that while areas with numerous entertainment venues may offer greater convenience, they may also be associated with higher levels of disruption to daily life. This trade-off means that areas with fewer entertainment venues—yet still adequate to meet basic needs—may have higher land prices.

#### 3.4.5. Economic factors result.

**Fig 12:**
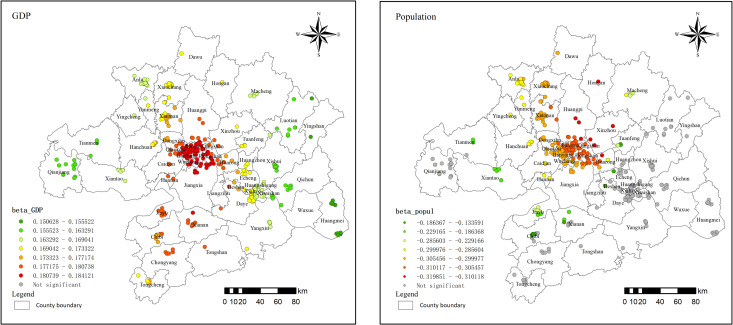
Economic factors regression coefficient distribution in 2021.

The regression coefficients for GDP range from 0.151 to 0.184, showing a positive global effect with overall statistical significance (Fig 12). In both the central and peripheral areas of the metropolitan region, increases in GDP contribute to higher residential land prices. Although the extent of this influence may vary across regions, the overall trend is consistent. Specifically, the positive effect is more pronounced in the north-south direction. In counties such as Hannan and Xian’an, proximity to the central urban area of Wuhan enhances economic spillover effects, driving economic activity and increasing the demand for construction land, which in turn raises residential land prices. As one moves eastward or westward, this positive relationship gradually weakens. In counties like Huangmei and Yingshan, characterized by mountainous terrain and designated as key national ecological function zones, economic development is relatively underdeveloped, and the influence on land price increases is weaker than in the central urban areas of Wuhan.

The regression coefficients for population range from −0.134 to −0.320, indicating a negative correlation with residential land prices, particularly in the northeastern part of the metropolitan area. In certain counties, such as Jiang’an and Wuchang, where the population is approaching saturation and new housing developments are limited, the increase in population does not further drive up land prices. The capacity of local infrastructure and employment opportunities struggles to keep pace with population growth, thereby limiting the stimulatory effect of population increases on the housing market. In such areas, land and real estate development tends to shift to emerging regions, such as Huangpi and Xinzhou. However, in these newly developed counties, the supply of new housing may exceed actual demand, disrupting the supply-demand balance and leading to a decrease in residential land prices instead of an increase.

#### 3.4.6. Other factors result.

**Fig 13:**
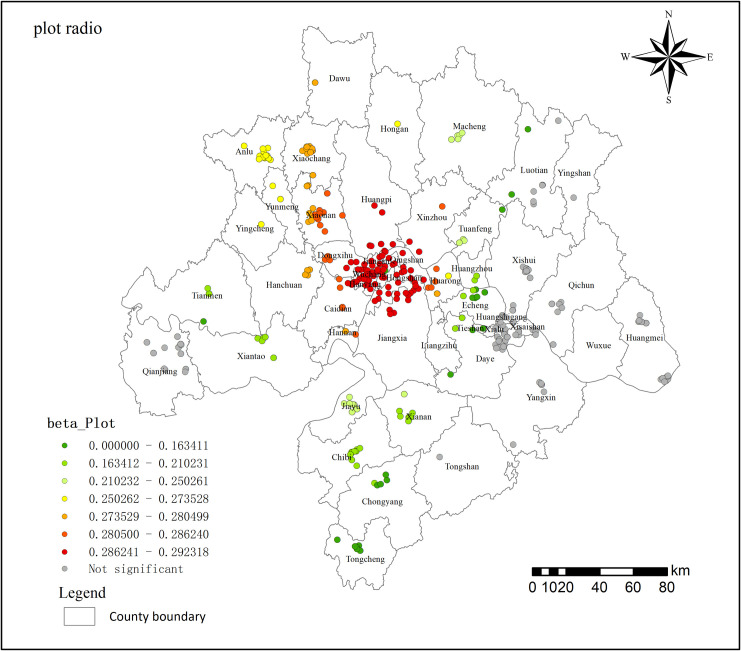
Other factors regression coefficient distribution in 2021.

The regression coefficient for the plot ratio in the Wuhan metropolitan area, with a value of 68.17%, demonstrates statistical significance, ranging from 0.018 to 0.292 (Fig 13). This indicates that as the plot ratio increases, residential land prices also rise. The influence of plot ratio within the study area exhibits a “single-center—stepped” pattern. In the metropolitan area, the available land for development in the central urban areas is limited. A higher plot ratio allows for more building area to be developed on a given plot of land, making developers more willing to pay higher land prices, thereby increasing the economic value of the land. Additionally, the Wuhan metropolitan area has proposed urban renewal initiatives that encourage high-density development, such as the redevelopment projects in Jiang’an and Qiaokou, further enhancing the competitiveness of high plot ratio land plots in the land market.

## 4. Discussion

This study explores the spatial differentiation and influencing factors of residential land prices in the Wuhan metropolitan area from 2015 to 2021. As urbanization accelerates within the metropolitan area, the continuous expansion of developed land has provided spatial resources for the development of residential properties. However, this expansion has also contributed to an imbalance in residential land prices across regions [55, 56]. Fueled by globalization and regional integration, the spatial structure of cities has evolved, leading to varying scales of metropolitan areas characterized by coordinated division of labor and mutual complementarity of interests between cities [57, 58]. A clear spatial discrepancy exists between the central urban areas, characterized by concentrated development and high housing prices, and peripheral regions, where residential land is more dispersed and prices are relatively low. This research analyzes the impact of economic, transportation, neighborhood, and plot ratio factors on residential land prices, demonstrating how elements such as population mobility, socioeconomic connections, public transportation accessibility, and the availability of public facilities have created conditions for inter-regional resource flow.

The empirical findings of this study indicate that residential land prices are relatively high in the central urban areas of Wuhan, while surrounding regions exhibit lower prices with smaller increases. There is a significant gap between the minimum and maximum land prices, pointing to evident regional imbalances in development. However, compared to 2015 and 2018, the situation has improved in 2021. As economic factors continue to spill over into surrounding cities, the imbalance in residential land prices near Wuhan has begun to ease, though this trend is most evident in counties closer to the city center. This pattern aligns with Wuhan’s efforts to strengthen integration with cities such as Ezhou, Xiaogan, and Huangshi, as well as the southeastern development axis of the metropolitan area.

Regarding the network structure, intercity land price connections have gradually strengthened. On the whole, the accessibility and stability of the residential land price network in the Wuhan metropolitan area have improved, although the strength of connections between spatial nodes presents room for enhancement. At the local level, the transmission role of node cities within the network development process has continued to increase. Surrounding areas near the city center have experienced better development, forming a stable hinterland layer that is continuing to expand. To further promote balanced regional development, policies should focus on accelerating intercity transportation connectivity, equalizing access to public services, and fostering urban-rural collaboration. Strengthening high-density connections between peripheral areas and the central urban districts, boosting talent attraction, capital inflows, and industrial settlement, and alleviating the functional load of the central city will support the development of smaller cities and towns within the metropolitan area [59]. The goal of achieving a balance between housing and employment should guide the development of a healthy residential land price market.

This study’s multi-scale geographically weighted regression (GWR) analysis is valuable for understanding regional variations in the driving factors behind residential land prices, suggesting that policy responses should be tailored to local contexts [60]. The regression results reveal that shopping malls are key geographical factors influencing spatial differences in land prices. It is recommended to strategically plan the distribution of modern commercial facilities to avoid excessive competition between the central city and surrounding counties, optimizing the placement of commercial nodes within various community life circles. Transportation factors also have a notable impact on core areas, and it is advisable to expedite the construction of intercity roads, address gaps in the metropolitan area’s transportation network, and create conditions that facilitate one-hour or shorter commute times. Parks and medical facilities positively affect land prices. Strategic planning to develop park clusters and fully utilize the ecological resources of the “Wuhan-Ezhou-Huangshi” region, including lakes, rivers, and the Dabie Mountains, would create more green open spaces for residents. Promoting shared development of medical resources across the Wuhan metropolitan area and enhancing public service levels in smaller cities and towns will help mitigate spatial imbalances in residential land prices. Plot ratio is a mandatory condition in land use rights transfers; government agencies can regulate residential land prices by adjusting plot ratios. The negative regression coefficient for population density in the Wuhan metropolitan area indicates an inverse relationship between population density and residential land prices. The “14th Five-Year Plan for Housing Development in Wuhan” has established plot ratio guidelines across different regions, aiming to manage residential land construction intensity scientifically, reduce population density, and improve housing quality and living environments.

This study also introduces innovations. Methodologically, by applying social network analysis, it investigates the network structural characteristics of residential land prices in the Wuhan metropolitan area, uncovering complex network relationships between counties and addressing the previous neglect of inter-city land price interactions and mobility. Additionally, the UNA tool, based on the Rhino platform, is used to measure the accessibility of facilities around residential sample points. Traditional spatial econometric models, such as Geographically Weighted Regression (GWR) and Spatial Autoregressive Models (SAR), have been widely employed in existing studies. This study, by applying the Multi-Scale Geographically Weighted Regression (MGWR) model, achieves a more precise characterization of spatial heterogeneity in the determinants of land prices than conventional OLS and GWR approaches, thereby enhancing predictive performance and model robustness. The findings contribute not only to the literature on land economics in the Wuhan Metropolitan Area but also offer theoretical insights for metropolitan regions across central China, including Chang-Zhu-Tan, Hefei, and Zhengzhou. Moreover, the results provide empirical support for policymakers in optimizing land resource allocation, improving the spatial distribution of urban-rural public services and infrastructure, and fostering integrated regional development.

This study has certain limitations. It focuses exclusively on the network characteristics of residential land prices derived from land transfer records between 2015 and 2021, without accounting for the potential impacts of surrounding completed real estate projects on sample land prices. In the analysis of influencing factors, a regression model was constructed from a micro-level perspective. Future research could incorporate policy-related factors, such as metropolitan integration policies, new district planning, and degrees of urban amalgamation, to further explore the formation mechanisms of residential land price networks. Additionally, expanding the study area to include peripheral regions of the metropolitan area and establishing these as a control group would allow for a more comprehensive evaluation. By comparing the annual variations in influencing factors between the experimental and control groups before and after policy interventions, the effects of regional coordinated development could be more accurately assessed.

## 5. Conclusion

Based on the residential land price data in the Wuhan metropolitan area in 2015, 2018, and 2021, this study employed social network analysis to investigate the spatiotemporal evolution characteristics of the metropolitan area network based on residential land prices, and applied a MGWR model to reveal its influencing factors. The main conclusions are as follows: (1) The land transfer residential land prices in the Wuhan metropolitan area show a certain hierarchical structure and polarization phenomenon, which has been somewhat alleviated in 2021. The regional residential land price level has significant spatial autocorrelation and local autocorrelation, the same agglomeration type shows obvious group distribution in space, and the high and high areas are mainly concentrated in the central urban area of Wuhan. (2) The networking of residential land prices in the Wuhan metropolitan area has strengthened but at a slow pace. Currently, it mainly relies on some nodal areas with significant regional differentiation. With the initial formation of the residential land price network in the Wuhan metropolitan area, the convenience between regions and stability of the network gradually improve. (3) Compared to OLS and GWR, the MGWR model more accurately measures the impact and spatial variability of variables on residential land prices. The contributing factors, ranked by their influence, are: shopping malls > secondary roads > population > plot ratio > parks and squares > medical facilities > GDP > entertainment venues. With the exception of population and entertainment venues, all other factors exert a positive influence on residential land prices to varying extents. To promote coordinated development in the metropolitan area, it is recommended to develop differentiated, hierarchical land supply and price regulation strategies tailored to the distinct characteristics of core, suburban, and fringe areas. By optimizing the efficiency of land resource allocation and guiding the rational distribution of population and industries, the urban agglomeration can be further developed.
